# Skimmin, a Coumarin from *Hydrangea paniculata*, Slows down the Progression of Membranous Glomerulonephritis by Anti-Inflammatory Effects and Inhibiting Immune Complex Deposition

**DOI:** 10.1155/2013/819296

**Published:** 2013-08-06

**Authors:** Sen Zhang, Hongqi Xin, Yan Li, Dongming Zhang, Jing Shi, Jingzhi Yang, Xiaoguang Chen

**Affiliations:** State Key Laboratory of Bioactive Substances and Functions of Natural Medicines, Institute of Materia Medica, Chinese Academy of Medical Sciences and Peking Union Medical College, Beijing 100050, China

## Abstract

Skimmin is one of the major pharmacologically active molecules present in *Hydrangea paniculata*, a medical herb used in the traditional Chinese medicine as an anti-inflammatory agent. In the current study, we attempted to investigate its renoprotective activity and underlying mechanisms in a rat model of membranous glomerulonephritis induced by cationic bovine serum albumin (c-BSA). Sprague-Dawley (SD) rats were divided into five groups, including normal control, model control, Mycophenolate Mofetil-treated group, and two skimming-treated groups (15 mg/kg and 30 mg/kg). Our research showed that treatment with skimmin significantly reduced the levels of blood urea nitrogen (BUN), urinary albumin excretion (UAE), and serum creatinine (Scr) as compared with model control after experimental induction of membranous glomerulonephritis (*P* < 0.01). Moreover, glomerular hypercellularity, tubulointerstitial injury, and glomerular deposition of IgG were less intense after skimmin treatment. By immunochemistry analysis, we demonstrated that skimmin could significantly inhibit interleukin-1**β** (IL1**β**) and IL-6 expression (*P* < 0.05), reduce the loss of nephrin and podocin, and suppress the infiltration of renal interstitium by CD3-positive T cell and CD20-positive B cell. These results suggest that treatment with skimmin can significantly improve renal function and suppress the IgG deposition as well as the development of glomerular lesions in a rat model of membranous glomerulonephritis.

## 1. Introduction

*Hydrangea paniculata *(family Hydrangeaceae) possesses anti-inflammatory and antipyretic properties. Water extract of branches and stems of *Hydrangea paniculata* has been used to treat numerous kidney diseases for decades. Skimmin that belongs to coumarin family is a common chemical compound present in many medical plants, such as *Aegle marmelos (*L*.) Correa *and *Adina cordifolia* [1–3]. Skimmin is also present in *Hydrangea paniculata*, accounting for approximately 55% of the total constituents in the water extract of its branches and stems.

In our previous study, we demonstrated a renal protective activity for skimmin in a rat model of streptozotocin-induced diabetic nephropathy [4]. Skimmin treatment significantly decreased plasma creatinine, improved creatinine clearance, and reduced the incidence of glomerulosclerosis and tubulointerstitial injuries in animals following diabetic nephropathy induction. Downregulation of expression of TGF*β*1 and TGF*β* receptor I [4], which play an important role in renal fibrosis of end-stage renal disease, is one of the possible mechanisms underlying the renoprotective activity of skimmin. 

Given the demonstrated activity of skimmin in protecting against renal injury in diabetic nephropathy, we hypothesize that skimmin may also have renoprotective effect in membranous nephropathy which is caused by immune complex localization in the subepithelial area of the glomerulus [5]. Inhibition of inflammatory mediators is one of the important target pathways for intervention of membranous nephropathy. In this study, we treated SD rats with cationic bovine serum albumin (C-BSA) to create an animal model of membranous glomerulonephritis and investigated the therapeutic effect of skimmin in protecting against renal injury in this model. Specifically, we assessed how skimmin modulated changes in the renal function and albuminuria, kidney cell morphology, IgG deposition, and expression of cytokines such as IL-1*β* and IL-6 induced by C-BSA. We also analyzed the expression of nephrin and podocin to assess the podocyte injuries and the renal interstitial infiltration by B cells and T cells to understand involvement of immune system in skimmin's renoprotective action in membranous nephropathy. 

## 2. Materials and Methods

### 2.1. Skimmin Preparation

Skimmin was provided by the Laboratory of Plant Natural Products, Institute of *Materia Medica*, Chinese Academy of Medical Sciences, which was prepared as follows. Air-dried stems of *H. paniculata* (5 kg) were powdered and extracted with H_2_O (2 × 20 L, each for 2 h). The H_2_O extract was passed through macroporous resin (D101, 5 kg) column and eluted with H_2_O (6 L), 30% EtOH (9 L), 70% EtOH (9 L), and 95% EtOH (8 L). The 30% EtOH fraction (A) was dried in vacuum, and the residue (120 g) was subjected to silica gel column chromatography (200–300 mesh, 1.5 kg) and eluted with CHCl_3_-MeOH-H_2_O (80 : 20 : 2, 6 L) to obtain eight fractions (Fr A-1~A-8). The precipitate was formed from the Fr A-4 after concentration and then filtered. The solid was repeatedly recrystallized in MeOH to yield skimmin (10.2 g). The purity of the compound was >95%, as determined by HPLC.

### 2.2. Preparation of C-BSA

To prepare C-BSA, crystallized unmodified BSA was chemically cationized according to Border's method [6]. An anhydrous ethylenediamine (EDA, Sigma-Aldrich, Germany) solution was prepared by mixing 67 mL of EDA and 500 mL of distilled water. The pH was adjusted to 4.75 with 350 mL of 6 M HCl at 25°C. After addition of 1.8 g 1-ethyl-[(3-dimethylaminopropyl)-carbodiimide hydrochloride] (EDC, Sigma-Aldrich, Germany), 5 g native BSA (Amresco, Solon, USA) dissolved in 25 mL of distilled water was added to the EDA solution. With continuous stirring the reaction was continued for 120 min, before being stopped by adding 30 mL 4 M acetate buffer. The product was dialyzed 48 h against distilled water at 4°C, lyophilized, and stored at −80°C.

### 2.3. Animals

Female SD rats, 10 weeks old and weighing 160–180 g, were obtained from the Institute of Laboratory Animal Science, Chinese Academy of Medical Sciences, Beijing, China. Rats were maintained under a 12 h light/dark cycle at 25°C and a humidity of 60 ± 10%. Experiments were performed in accordance with the institutional regulations on the use of experimental animals. Experimental design followed the methods of Border et al. [6] and Mirshafiey et al. [7]. A total of 50 rats were randomly divided into five groups: normal vehicle treatment (N group), model (M group), low skimmin treatment at 15 mg/kg body weight (L group), and high skimmin treatment at 30 mg/kg body weight (H group). Mycophenolate Mofetil (MMF group) at 20 mg/kg was used as a positive control. Rats in the M, MMF, L, and H groups were injected subcutaneously with 0.5 mg incomplete Freund's adjuvant at day 1 to prevent autoimmunity, followed by injection of C-BSA (50 mg/kg, administered at 10 mg/mL in 0.01 MPBS, pH 7.4) through the tail vein every other day from days 8 to 36 to induce membranous glomerulonephritis. Rats in the N group were injected with saline every other day during the same time. From days 37 to 66, rats in the MMF, L, and H groups received daily intragastric MMF and skimmin (MMF group: 20 mg/kg; L group: 15 mg/kg; H group: 30 mg/kg), while rats in the N and M groups received daily intragastric saline. All animals were sacrificed on day 66.

 All the animal experiments were approved by the Ethics Committee of Laboratory Animals of Peking Union Medical College (Beijing, China); the protocol was approved on, June, 15, 2011 (approval number 002463).

### 2.4. Blood and Urine Chemistry

Before sacrifice, blood was sampled from all animals through the eyes under anesthesia with diethyl ether. 24-hour urine collections were performed in each animal after placement in metabolic cage the day before blood sample collection. BUN, Ucr, and Scr were measured with the commercial kits (BHKT clinical reagent Co. LTD, Beijing, China). Creatinine clearance ratio (Ccr) was calculated according to the following formula:

(1) Ccr=Urinary  creatinine  (mg/mL)   ×urine  volume  (mLkg)creatinine  in  plasma  (mgmL).

See [8]. 

### 2.5. Competitive ELISA of Rat Urinary Albumin Excretion

Urinary albumin excretion was calculated from albumin concentration of urine samples collected in metabolic cage for 24 h. Indirect ELISA was used to quantitatively measure urinary albumin concentrations. Briefly, the 96-well plate was coated with rat serum albumin (RSA, 1 *μ*g/mL, 50 *μ*L, Sigma, MO, USA) and then blocked. Following a thorough washing, 50 *μ*L PBS-diluted rat urine or RSA standards (0–10 *μ*g/mL) were added to the wells and incubated. Then 50 *μ*L HRP conjugated sheep anti-rat albumin antibody (0.1 *μ*g/mL, Bethyl, TX, USA) was added and the color was developed by TMB (3,3′,5,5′′-tetramethylbenzidine). Urinary albumin excretion was expressed as mg/day.

### 2.6. Histological Examination

Kidney specimens were processed by light and immunofluorescence microscopic examination. For the light microscopy, the right kidneys from each animal were fixed in 10% phosphate-buffered formalin solution and embedded in paraffin. Sections of 2 *μ*m thickness were cut and stained with hematoxylin and eosin (HE) and periodic acid-Schiff (PAS). To evaluate the glomerular hypercellularity, at least 10 glomeruli were examined for each animal, the number of cells in each glomeruli (including endothelial cells, mesangial cells, and podocytes) was counted and average number was calculated; meanwhile, the incidence of glomerular basement membrane thickening or mesangial proliferation among 100 glomeruli was calculated too. 

To assess the tubulointerstitial damage, a semiquantitative method of renal histology using a grading scale of 0–4 was applied: 0: normal; 1: lesions in <25% of the area; 2: lesions in 25% to 50% of the area; 3: lesions in >50% of the area; and 4: lesions involving the entire area [9, 10]. Tubular atrophy, dilation, casts, interstitial inflammation, and fibrosis were assessed in 10 kidney fields at a magnification of ×100. 

For immunofluorescence microscopy, tissue blocks from the left kidney were instantaneously frozen in n-hexane precooled to −70°C, and 4 *μ*m cryostat sections were stained with fluorescein isothiocyanate- (FITC-) conjugated anti-rat IgG. The degree of deposition of immune complex was calculated quantitatively on the basis of the staining intensity and distribution. 

### 2.7. ELISA Analysis of IL-1*β* and IL-6 in Plasma

Plasma levels of IL-1*β* and IL-6 were measured by with ELISA kit following the manufacturer's instructions (R&D systems, USA). Briefly, a monoclonal antibody specific for IL-1*β* or IL-6 was precoated onto a microplate. The standards and test samples were then pipetted into the wells to allow binding of IL-1*β* or IL-6 to the immobilized antibodies. After washing away any unbound substances, an enzyme-linked polyclonal antibody specific for IL-1*β* or IL-6 was added to the wells. Following removal of unbound antibody-enzyme reagent through washing, a substrate solution was added to the wells and color was developed. The optical density of each well was measured at the wave length of 450 nm or 510 nm on a microreader (S190, Molecular Devices, USA). The concentration of IL-1*β* or IL-6 was accordingly calculated. 

### 2.8. Immunohistochemistry

The expression of IgG, IL-1*β*, and IL-6 in kidney was assessed by the streptavidin-peroxidase-biotin (SP) immunohistochemical method [11–13]. The kidney tissue sections (4 *μ*m) were made and were mounted onto immunohistochemical slides. After dehydration with xylene and alcohol, the antigen retrieval was accomplished. Sections were then incubated in a blocking solution of 10% normal goat serum diluted in PBS and incubated with polyclonal rabbit anti-rat IgG (Abcam, USA), IL-1*β* (Abcam, USA) and IL-6 (Abcam, USA), antibodies (1 : 200) for 2 h and then incubated for 10 min at room temperature with biotinylated goat anti-rabbit secondary antibody (1 : 500). After that, sections were exposed to streptavidin-biotin complex conjugated to HRP. Signals were then visualized with diaminobenzene (DAB) and observed at 200x magnification under a light microscope (Leica, Germany). Signals in three random fields on each slide were quantitated using Image-Pro Plus software (Bethesda, MD, USA). Results were expressed as percentage of positive area per glomerulus.

To understand whether skimmin had protective effect on podocytes, we evaluated the expression of nephrin and podocin in glomeruli by immunohistochemistry following the similar procedure described before. Monoclonal antinephrin (Santa Cruz, USA) and monoclonal anti-podocin (Abcam, USA) were used, respectively. 

To assess the distribution of B cell and T cell infiltration into the renal interstitium, immunohistochemistry with an anti-CD3 (Abcam, USA) antibody and an anti-CD20 antibody (Santa Cruze, USA) was used. The same immunohistochemistry procedures as mentioned before were followed. 

### 2.9. Statistical Analysis

Data were expressed as the means ± standard deviation (S.D.) and analyzed by One-Way Analysis of Variance (One-Way-ANOVA) followed by and post hoc Bonferroni test. Pearson's correlation analysis was used to determine the relationship between IL-1*β* or IL-6 with the values of IgG deposition and the degree of tubulointerstitial damage. Difference was considered significant when *P* < 0.05 and *P* < 0.01. 

## 3. Results

### 3.1. Effect of Skimmin on Renal Function

As shown in Table 1, C-BSA markedly caused severe deterioration of renal functions compared to the normal control. The average level of albuminuria increased from 0.22 (mg/day) to 5.64 (mg/day) dramatically (normal versus model, *P* < 0.01). Skimmin treatment at 15 mg/kg and 30 mg/kg decreased average albuminuria level by 13.5% (*P* < 0.05), and 36.0% (*P* < 0.05), respectively. MMF treatment at 20 mg/kg also decreased albuminuria level by 30.4% (*P* < 0.05). In the model group, levels of Scr and BUN were significantly higher than those in the normal control group (Table 1). Skimmin (30 mg/kg) treatment reduced BUN by 42.7% (*P* < 0.05) and Scr by 55.7% (*P* < 0.05), respectively, compared to model group. Compared to the model group, skimmin treatment (30 mg/kg) increased the Ucr by 51.6% (*P* < 0.05) and increased Ccr by 67.8% (*P* < 0.05). These biochemical analysis results indicated that skimmin at a dose of 30 mg/kg was effective in the protection against renal injury. 

### 3.2. Morphological Changes

**Figure 1:**
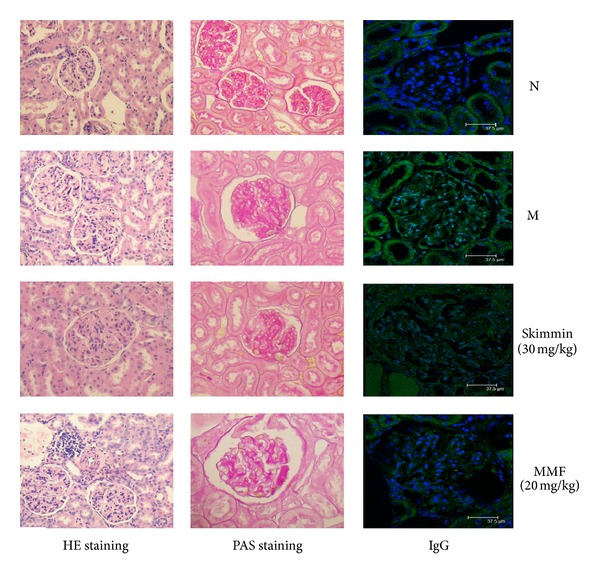
Representative images of HE-stained and PAS-stained kidneys and immunofluorescent microscopy of IgG deposition (100x).

Shown in Figure 1 are light microscopic graphs (100x) of renal glomeruli and tubules in different groups of animals. As compared to vehicle controls (Normal group), C-BSA treatment produced numerous slightly enlarged renal glomeruli, capillary stenosis, and thickened capillary walls with scattered short subepithelial basement membrane projections (Model group). Treatment with 15 or 30 mg/kg skimmin reduced these C-BSA-induced morphological changes. The incidences of glomerular hypercellarity, glomerular basement membrane thickening or mesangial proliferation, and tubulointerstitial injuries were all significantly reduced in the skimmin treatment groups than in the model control (*P* < 0.01, Table 2). 

Immunofluorescent microscopic investigation of glomeruli revealed deposits of immune complexes in the mesangial areas and along the capillary walls of all animals. But the deposition was significantly less intense in skimmin and MMF treated animals than that in the vehicle controls; at the dose of 30 mg/kg, skimmin reduced the IgG positive staining area by 41% compared to model group (*P* < 0.01, Figure 1, Table 2). 

### 3.3. Expression of IgG, IL-1*β*, and IL-6 by IHC and ELISA

**Figure 2:**
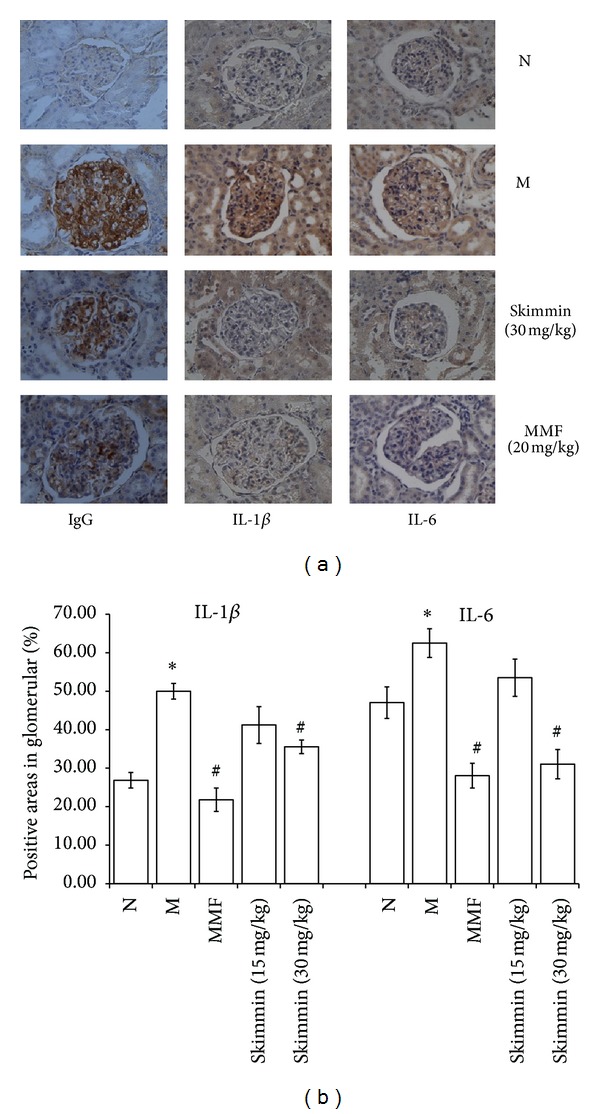
Immunohistochemical staining, showing the expression of IgG, IL-1*β*, and IL-6 (200x, designated by percentage of positive areas in glomerulus). *P < 0.05; **P < 0.01, versus normal control; ^#^ *P* < 0.05, ^##^ *P* < 0.01, versus model control, by One-Way-ANOVA followed by post-Hoc test.

As shown in Figure 2, immunohistochemistry demonstrated that C-BSA induction markedly increased the IgG accumulation and the expression of IL-1*β* and IL-6 (M group) in glomeruli compared with vehicle controls (N group) (*P* < 0.05). The IHC results on IgG accumulation in glomeruli were consistent with fluorescent microscopic results. Skimmin dose dependently decreased the expression of IL-1*β* and IL-6 in glomeruli (*P* < 0.05). However, skimmin at 30 mg/kg was less potent than MMF at 20 mg/kg.

**Figure 3:**
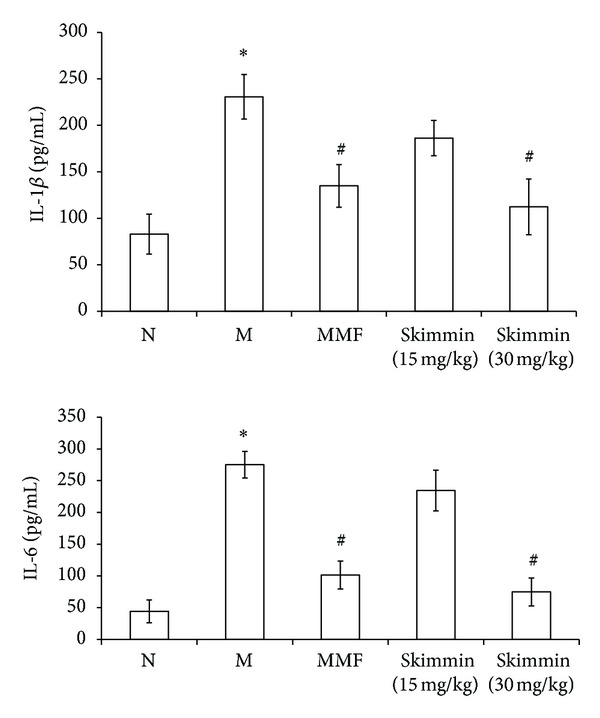
ELISA presented IL-1*β* and IL-6 levels in plasma of skimmin-treated rats were reduced significantly than the model control rats. *P < 0.01 as compared with the normal rats; ^#^ *P* < 0.05 as compared with model control rats.

ELISA analysis showed that plasma concentrations of IL-1*β* and IL-6 were significantly decreased in the high (30 mg/kg) skimmin and MMF treatment groups, as compared with the model control groups (*P* < 0.05, Figure 3).

### 3.4. Changes in Nephrin and Podocin Expression in the Glomeruli

**Figure 4:**
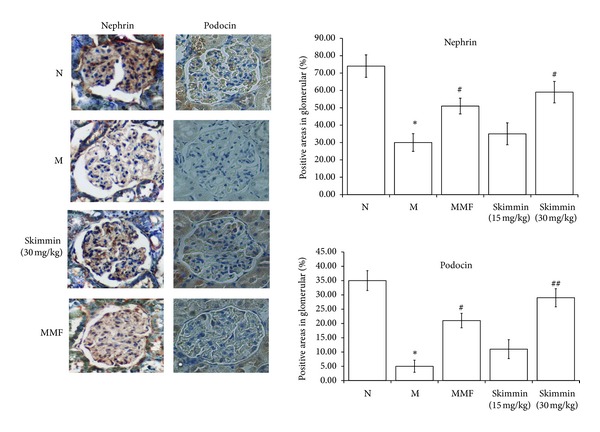
Expression and quantitation analysis of podocin and nephrin in the glomeruli, (200x, designated by percentage of positive areas in glomerulus). *P < 0.01 as compared with the normal rats; ^#^ *P* < 0.05, ^##^ *P* < 0.01 as compared with model control rats.

Changes in expression of nephrin and podocin related to podocyte injuries were examined by immunohistochemical staining. As shown in Figure 4, immunoreactivity for nephrin and podocin in the glomerular regions of rats in the model group was significantly decreased, and skimmin (30 mg/kg) and MMF treatment effectively ameliorated the C-BSA-induced aberration in the expression of nephrin and podocin (*P* < 0.05). 

### 3.5. CD20 Positive B Cell and CD3 Positive T Cell Infiltration of Interstitium

**Figure 5:**
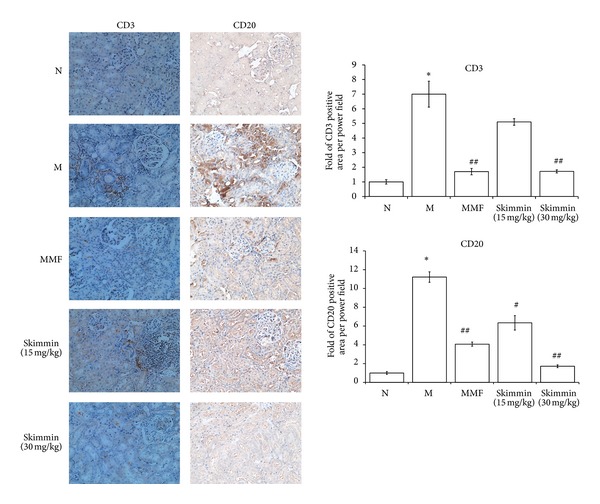
Representation and quantification of the infiltrates by digital morphometry, determined as the percentage of area staining positive. Arrows refer to CD3 positive T cells, 200x. *P < 0.01 as compared with the normal rats; ^#^ *P* < 0.05, ^##^ *P* < 0.01 as compared with model control rats.

To further characterize lymphocyte infiltration of the renal interstitium, we performed immunohistochemistry to detect the distribution of CD3 mature T lymphocyte and CD20 mature B lymphocyte in the interstitium. Obvious focal or diffuse interstitial CD20 positive B cell infiltration was seen in the model group but not in the normal control group; in the MMF and skimmin (30 mg/kg) treatment groups, weak and moderate CD20 positive staining was detected, suggesting both MMF and skimmin were able to reduce the CD20 mature B cell infiltration significantly (*P* < 0.05, Figure 5). 

Under microscopic view, small aggregates of CD3 positive T-cells were detectable in the renal interstitium in the model group (Figure 5). In the MMF and skimmin (30 mg/kg) treatment groups, positive CD3 cells was less aggregated were found, and most of them were singly present in the interstitium, suggesting that both MMF and skimmin could ameliorate the CD3 T cell infiltration (*P* < 0.05, Figure 5).

### 3.6. Correlation between IL-1*β* and IL-6 with IgG Deposition and the Degree of Tubulointerstitial Damage

**Figure 6:**
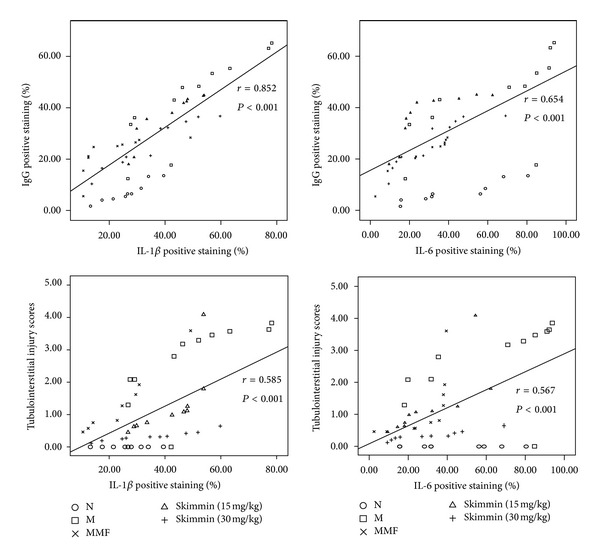
Correlation between IL-1*β* and IL-6 with the values of IgG deposition and the degree of tubulointerstitial damage.

Finally, we examined the correlation of IL-6 and IL-1*β* as inflammatory markers with the values of IgG deposition and the degree of tubulointerstitial damage. A positive correlation between IgG deposition and levels of IL-6 or IL-1*β* was demonstrated in all animals (*P* < 0.01); similar correlation was also observed between the degree of tubulointerstitial damage and levels of IL-6 or IL-1*β* (Figure 6). Together, these results suggested that changes in IL-6 or IL-1*β* concentrations are closely related to the pathogenesis of membranous glomerulonephritis. 

## 4. Discussion 

Membranous glomerulonephropathy is the most frequent cause of nephrotic syndrome in adults [14, 15], but currently there is no effective medicines. The major pathological characteristic about membranous glomerulonephropathy is the presence of subepithelial immunoglobulin-containing deposits along the glomerular basement membrane, which was found in the model group in the current study. In the clinical practice, angiotensin converting enzyme (ACE) inhibition or angiotensin receptor blockade is still most common strategy against membranous nephropathy or, rather, more aggressive treatment using glucocorticoids and alkylating agents [5]. Recent progress in the molecular pathways of inflammation and immunologic regulation holds the promise of offering futuristic alternatives and/or supplements to the standard regimen, such as inhibition of proinflammatory mediators and T and B cell proliferation and activation; besides, proteinuria control and suppressing activation of TGF*β*-smad pathway are also potential alternative strategies. 

The present study evaluated the therapeutic effect of skimmin on glomerulonephritis in a rat model of C-BSA induced membranous glomerulonephritis. C-BSA administration dramatically increased BUN, Scr, and albuminuria, caused morphologically structural changes and increased IgG deposition and expression of some cytokines, such as IL-1*β* and IL-6. Skimmin treatment suppressed all these abnormalities significantly. 

In the current study, skimmin treatment, especially at a high dose, significantly alleviated the tubulointerstitial injuries (*P* < 0.01). It was able to reduce the albumin content in the urine significantly. Previous studies have demonstrated that, under proteinuric conditions, albumin is the major protein in the ultrafiltrate and nephrotic urine, which plays an active role in the pathogenesis of chronic tubulointerstitial damage [16–18]. We assume that albuminuria reduction is one of mechanisms by which skimmin alleviates tubulointerstitial injuries. 

Podocytes function to maintain the permeability of the glomerular filtration barrier; their dysfunction and depletion contribute greatly to albuminuria [19, 20]. Studies have established an association between the development of progressive kidney disease and podocyte failure [21, 22], as seen in gene mutations of podocyte-specific proteins leading to nephrotic syndrome, renal injury, and failure [23–27]. Several molecules synthesized by podocytes, such as podocin, nephrin, CD2-associated protein (CD2AP), NcK, ZO-1, and actinin [27, 28], play key roles in the maintaining of integrity of the glomerular filtration barrier. In the current study, low expression of podocyte biomarkers (nephrin and podocin) was observed in the glomeruli of animals in the model group, in consistency with significantly higher albuminuria in the model group. Skimmin treatment significantly inhibited the loss of nephrin and podocin expression in a dose dependent manner. This suggests that skimmin protection of podocytes may partially contribute to its albuminuria-lowering activity. 

The effect that skimmin suppresses glomerulonephritis might be also attributable to its ability to inhibit the expression of some pro-inflammatory cytokines. Both IL-1*β* and IL-6 are important cytokines which are essential mediators of immune response and inflammatory reactions in patients with chronic renal failure [29]. In the current study, by correlation analysis, we prove that IL-1*β* and IL-6 expressions are positively correlated with tubulointerstitial injuries and IgG deposition. Possibly, skimmin may exhibit its anti-inflammatory effect and subsequent renoprotection through inhibiting IL-1*β* and IL-6. 

Focal or diffuse interstitial B cell infiltration was detected by immunostaining with anti-CD20 antibody in the model group. By contrast, this infiltration was less significant in the skimmin treatment groups. CD3 positive T cell infiltration was much weaker than CD20 B cell infiltration in the renal interstitium in the current study. B cell and T cell infiltration is important pathological characteristics in membranous nephritis [30, 31]. By suppressing T cell and B cell infiltration, skimmin may ameliorate the severity of inflammation and slow down the development of renal dysfunction. 

Membranous glomerulonephritis is an antibody-mediated disease induced by deposits of immunoglobulins and complement components on the subepithelial layer of the glomerular capillary wall [32]. This immune deposition promotes injury to the glomerular filtering barrier, proteinuria, and eventual renal failure [33]. Results in experimental membranous glomerulonephritis have shown that the inhibition of B cell function is associated with beneficial effects on proteinuria [34] and human studies clearly demonstrated that the inhibition of B cells with alkylating agents induces remission of the nephrotic syndrome [35]. In B cell development, IL-6 has been shown to induce terminal maturation of B lymphocytes into antibody producing plasma cells [36, 37]. Immunohistochemistry results in the present study suggest that skimmin may inhibit B cell maturation and function by decreasing IL-6 levels in kidneys, leading to beneficial effect on membranous glomerulonephritis. 

MMF has been used successfully as an immunosuppressive medication in lupus nephritis and other primary glomerular diseases [38]. Lots of experimental animal models of nephritis and clinical trials have proved that MMF could slow down the progress of several glomerular diseases, partially from inhibiting both mesangial cell proliferation and lymphocyte migration into renal tissue [39, 40]. Such inhibition results from impaired glycosylation, which, in turn, leads to alterations in adhesion molecule functioning [41]. In the current study, as a positive control, at 20 mg/kg dosage, MMF shows similar beneficial effect on membranous nephropathy as skimmin, which gives us more confidence to further explore the skimmin's potential usage in the clinical treatment. 

## 5. Conclusions

In summary, despite the use of *H. paniculata* as an anti-inflammatory agent in Chinese traditional medicine for long, its renoprotective activity and the underlying mechanism in the membrane glomerulonephritis have not been established. This study is the first to report the effects of skimmin, a major constituent present in *H. paniculata*, on IL-1*β* and IL-6 protein expression in membranous glomerulonephritis. Our observations suggest that skimmin is a promising therapeutic agent for glomerulonephritis. 

## Figures and Tables

**Table 1:** Renal function in the membranous glomerulonephritis model rats after treatment with the skimmin for 30 days.

Index	Sham control	Model control	Groups		
			MMF (20 mg/kg)	Skimmin (15 mg/kg)	Skimmin (30 mg/kg)
*N*	10	10	10	10	10
Body weight (g)	311.25 ± 18.15	293.65 ± 28.46	287.00 ± 28.79	284.50 ± 28.79	281.70 ± 20.00
Urine creatinine (mM)	4.34 ± 1.58	2.87 ± 1.17*	3.02 ± 1.74	3.00 ± 1.51	4.35 ± 1.10^#^
Blood urea nitrogen (mM)	7.53 ± 1.40	21.08 ± 17.30*	16.21 ± 10.13	22.25 ± 11.80	12.08 ± 10.09^#^
Serum creatinine (mM)	0.16 ± 0.10	1.22 ± 1.31*	0.98 ± 1.17	1.24 ± 0.91	0.54 ± 0.87^#^
Creatinine clearance (mL/min)	2.57 ± 0.01	1.43 ± 0.03*	1.26 ± 0.02	0.75 ± 0.05	2.40 ± 0.07^#^
Urinary albumin excretion (mg/day)	0.22 ± 0.15	5.64 ± 3.01**	3.93 ± 2.23^#^	4.88 ± 2.96^##^	3.61 ± 2.14^##^

*P < 0.05, **P < 0.01, versus normal control; ^#^ *P* < 0.05, ^##^ *P* < 0.01, versus model control, by One-Way-ANOVA followed by post hoc test.

**Table 2:** Light microscopic and immunofluorescent findings of kidney histological lesions.

Groups	*N*	Cell number per glomerulus	Case of mesangial proliferation (per 10 glomeruli)	Tubulointerstitial injury scores	IgG deposition (% positive area in glomerulus)
N	10	72.92 ± 12.52	0.30 ± 0.48	0	8.17 ± 3.52
M	10	99.28 ± 18.83**	4.73 ± 1.43**	1.20 ± 1.25**	45.86 ± 7.97**
MMF (20 mg/kg)	10	103.04 ± 21.69	3.40 ± 2.46	1.20 ± 0.76	21.99 ± 6.64^##^
Skimmin (15 mg/kg)	10	110.92 ± 29.38	1.10 ± 0.88^##^	1.27 ± 1.56	36.17 ± 4.76^##^
Skimmin (30 mg/kg)	10	81.80 ± 17.92^##^	1.20 ± 1.34^##^	0.33 ± 0.47^#^	26.00 ± 5.376^##^

Note: **P < 0.01, versus normal control; ^#^ *P* < 0.05, ^##^ *P* < 0.01, versus model control, by One Way-ANOVA followed by post hoc test.
